# The relationship between positive future thinking, entrapment, defeat and death-related mental imagery in individuals with and without a suicidal history: an experimental study

**DOI:** 10.3389/fpsyg.2025.1574315

**Published:** 2025-06-04

**Authors:** Gonca Köse, Jonathan J. Evans, Rory C. O’Connor

**Affiliations:** ^1^Suicidal Behaviour Research Laboratory, School of Health and Wellbeing, University of Glasgow, Glasgow, United Kingdom; ^2^Department of Psychology, Akdeniz University, Antalya, Türkiye; ^3^School of Health and Wellbeing, University of Glasgow, Glasgow, United Kingdom

**Keywords:** positive future thinking, future thinking, suicide risk, cognitive factors, IMV model

## Abstract

**Background:**

Although there is growing evidence that impaired positive future thinking is associated with suicide risk, the relationship between positive future thinking and suicidal thoughts or suicidal behaviours has yet to be completely understood. Therefore, this experimental study explored the role of positive future thinking in those with different suicidal histories.

**Methods:**

Fifty-three adults were recruited to a study, which included completing a range of self-reported measures (suicidal history, suicide ideation, defeat, entrapment, depression, and death-related mental imagery) and experimental tasks (verbal fluency or cognitive performance task, positive future thinking task and positive-and negative-mood induction). The study compared 30 individuals who had a history of suicidal thoughts/behaviours and 23 with no suicidal history in terms of positive future thinking before and after a negative mood induction and on established psychological markers of suicide risk (e.g., depression, defeat, entrapment, and death-related mental imagery).

**Results:**

Participants with a history of suicidal thoughts/behaviours reported significantly fewer positive future thoughts (PFT) than participants without past suicidal thoughts/behaviours. Mean scores for PFT from pre-to post-negative mood induction decreased significantly in the participants with a history of suicidal thoughts/behaviours but not in those without a suicidal history. Individuals with a history of suicidal thoughts/behaviours reported significantly higher levels of death-related mental imagery, depression, entrapment, and defeat compared to those without past suicidal thoughts/behaviours.

**Conclusion:**

This study provides experimental evidence that positive future thinking is implicated in the suicidal process. In addition, positive future thinking is affected by a negative mood induction, especially in individuals with a suicidal history. Changes in positive future thinking could be usefully assessed in clinical contexts.

## 1 Introduction

Suicide is a major public health concern, with more than 720,000 individuals dying by suicide every year worldwide and at least 20 times that number attempting suicide (World Health Organization, 2024). It is well known that the factors involved in the emergence of suicidal thoughts and suicidal behaviours are diverse, including psychological, demographic, clinical, environmental, biological, social, and cultural aspects and their interactions (Williams, 2001; Turecki et al., 2019). However, suicides are preventable with timely, evidence-based, and often low-cost interventions and/or effective and comprehensive multisectoral suicide prevention strategies (World Health Organization, 2024). In this study, we have focused on a selection of psychological factors (i.e., positive future thinking, entrapment, defeat, depression, and death-related mental imagery) that are implicated in the pathways to suicidal thoughts and suicidal behaviours. A modest number of studies has investigated the extent to which positive future thinking, defined as specific positive expectancies about the future, is associated with suicidal thoughts and suicidal behaviours (MacLeod and O'Connor, 2018; Walsh, 1993; Williams et al., 1996; Williams et al., 2008; Hales et al., 2011; Rasmussen et al., 2010). Taking these studies as a whole, the absence of positive thoughts about the future (i.e., positive future thinking, PFT, for example, ‘I am looking forward to spending time with my family tomorrow’) rather than the over-representation of negative thoughts about the future (i.e., negative future thinking, NFT, for example, ‘I am worried about getting the results of a test next month’) is associated with suicide risk (i.e., suicidal thoughts and suicidal behaviours) independent of depression, verbal fluency or cognitive performance (MacLeod et al., 1997; O’Connor et al., 2000; Sargalska et al., 2011). When this finding was first reported, it was noteworthy as it suggested that hopelessness in the context of suicide risk, characterised by the absence of positive future thinking, is more pernicious than hopelessness characterised by the presence of negative future thinking (MacLeod et al., 2005; MacLeod and O'Connor, 2018). More recently, difficulty imagining discrete actions in the context of positive future thinking has also been shown to be associated with suicidal ideation in adolescents (Cha et al., 2024) as has the novelty of the positive future thinking (Nam and Cha, 2024). However, there have been a few exceptions to these findings (Pollak et al., 2021; O’Connor et al., 2015). Specifically, in an adolescent sample, Pollak et al. (2021) found that the relationship between defeat/entrapment and suicidal ideation was strongest among adolescents with higher levels of positive future thinking. Whereas, in O’Connor et al. (2015), although low levels of positive future thinking were associated with suicidal ideation, high levels of intrapersonal positive future thinking predicted suicide attempts.

In addition, it is also not clear what happens to PFT in those with and without a suicidal history when they experience low mood (O’Connor and Williams, 2014). In one previous study, Williams et al. (2008) found that positive future thinking decreased after a negative mood induction in those with a suicidal history and that the changes were not accounted for by depression but were associated with hopelessness/suicidal ideation cognitive reactivity. However, the extent to which the decrease in positive future thinking is linked to suicidal history is unclear as this study had no control group.

This study, therefore, aimed to address the limitations of previous research by experimentally manipulating negative mood to investigate its effect on PFT in individuals with and without a lifetime history of suicidal thoughts/behaviours (hypotheses 1 and 2). In addition, we aimed to replicate previous studies (see Souza et al., 2024; O'Connor and Nock, 2014) that have explored the extent to which those with and without a suicidal history differ in established suicide risk factors (i.e., depression), including those derived from the IMV model (i.e., entrapment, defeat, and death-related mental imagery; hypothesis 3).

We tested the following hypotheses:

1. Individuals with a history of suicidal thoughts and/or suicidal behaviours will generate fewer positive future thoughts (PFT) compared to those without a history of suicidal thoughts or suicidal behaviours.
2. After a negative mood induction, it is expected that the level of PFT will decrease more in the group with a lifetime history of suicidal thoughts and/or suicidal behaviours compared to a control group (without a suicidal history), independent of baseline depression.
3. The likelihood of being in the group with a history of suicidal thoughts and/or suicidal behaviours (versus not) will be associated with levels of depression, entrapment, defeat, and death-related mental imagery.

## 2 Materials and methods

### 2.1 Participants

Fifty-three adults took part in the study. Individuals with a lifetime history of suicidal thoughts and/or suicidal behaviours (*n* = 30; 16 participants reported a suicide attempt history) and individuals without a suicidal history (*n* = 23) aged 18 years or older and living in Scotland comprised the study sample.

### 2.2 Measures

#### 2.2.1 Self-reported measures

Participants completed the following measures:

*Demographics*. Information about age, sex, gender, ethnicity, marital status, level of education, employment, sexual orientation, medication use, and history of psychiatric illness was obtained.

*Suicidal ideation*. Suicidal ideation was assessed via the eight-item Suicide Ideation subscale of the Suicide Probability Scale (SPS; Cull and Gill, 1989) which is a valid and reliable measure of suicide risk (Tatman et al., 1993; Go et al., 2000). Its score ranges from zero to 24 and it assesses various thoughts of suicide, such as ‘I feel the world is not worth continuing to live in’ and participants indicate how each statement applies to them on a 4-point scale from ‘none of the time’ (0) to ‘most or all of the time’ (3). Higher scores demonstrate higher levels of suicidal ideation. The measure showed excellent internal consistency in the current study (Cronbach’s *α* = 0.90).

*Suicidal History*. This was measured through two items from the Adult Psychiatric Morbidity Survey (McManus et al., 2016). Suicidal ideation and suicide attempts, respectively, were assessed as follows: (i) ‘Have you ever seriously thought of taking your life, but not actually attempted to do so?’; (ii) ‘Have you ever made an attempt to take your life, by taking an overdose of tablets or in some other way?’ Those who answered yes to either of these questions were allocated to the suicidal history group.

*Depression*. The Patient Health Questionnaire (PHQ-9; Cameron et al., 2008) is a screening tool for depressive symptoms. Items one to eight are for the assessment of depressive symptoms, and the last item (item nine) measures suicide ideation. To minimise contamination with the Suicide Probability Scale (SPS; Cull and Gill, 1989), we used the first eight items as a measure of depressive symptoms in this study. Participants respond via a 4-point Likert-type scale from (0) ‘not at all’ to (3) ‘nearly every day’ and scores range from zero to 24 when item nine was excluded. Based on the current study, excellent internal consistency was detected for this measure (Cronbach’s *α* = 0.91 for PHQ-8).

*Entrapment.* The Entrapment Scale (Gilbert and Allan, 1998) assesses both internal entrapment, involving six items about one’s own thoughts and feelings (e.g., ‘I feel powerless to change myself’) and external entrapment, including 10 items assessing feeling trapped by external situations (e.g., ‘I have a strong desire to escape from things in my life’). In this study, we used the 4-item Entrapment Scale–short form (De Beurs et al., 2020) which is an empirically derived brief version of the entrapment scale (using items four, five, 14, and 16 of the original scale). Responses are collected on a 5-point Likert-type scale from ‘not at all like me’ (0) to ‘extremely like me’ (4). Higher scores indicate a greater sense of entrapment, and scores for the 4-item Entrapment Scale Short-Form (E-SF; De Beurs et al., 2020) range from zero to 16, for both internal and external entrapment subscales scores range from zero to eight. Both internal and external entrapment sub-scales were found to have high levels of reliability in both student and clinical populations (>0.85; Gilbert and Allan, 1998). In this study, excellent internal consistencies for internal entrapment subscale (Cronbach’s *α* = 0.93), external entrapment subscale (Cronbach’s α = 0.89) and the total short scale (Cronbach’s *α* = 0.92) were found.

*Defeat*. The Defeat Scale (Gilbert and Allan, 1998) is a 16-item measure of an individual’s perceived struggle or loss of social rank (e.g., ‘I feel that I have not made it in life’), which has been found to be related to low psychological health. Participants respond via a 5-point Likert-type scale from (0) ‘never’ to (4) ‘always’ and scores range from zero to 64. Higher scores show greater levels of defeat, and this measure has been found to have high internal consistency in the general population (i.e., 0.94 in the student population; Gilbert and Allan, 1998). In this study, we used four items from a short form of the original Defeat Scale (Griffiths et al., 2015) using a 5-point Likert-type scale from (0) ‘never’ to (4) ‘always’ and scores range from zero to 16. The measure showed excellent internal consistency in the current study (Cronbach’s *α* = 0.93).

*Death-related Mental Imagery*. This measure developed by Holmes et al. (2007) includes asking eight questions to establish the frequency with which participants imagine death-related imagery when they are feeling down or distressed, involving engaging in suicidal behaviours (e.g., images of yourself planning or preparing to harm yourself or make a suicide attempt, and images of what might happen to other people if you died). Participants respond via a 5-point Likert-type scale from (0) ‘none of the time’ to (4) ‘all of the time’. Scores range from zero to 32. The measure showed good internal consistency in this study (Cronbach’s α = 0.83).

#### 2.2.2 Experimental tasks

*Verbal Fluency*. The Verbal Fluency Task (FAS) is a standard measure of cognitive performance or verbal fluency which is used as a control task in this study (Lezak, 1995). It includes asking study participants to write as many words as they can think of beginning with each of the three letters: F, A, S. Participants are given 1 min for each letter, and the three letters are presented in a fixed order, F, A, S. The mean number of acceptable words generated for each letter is calculated. This task was administered just before the positive future thinking task.

*Positive Future Thinking Task*. Positive Future Thinking (PFT) was assessed following MacLeod et al. (1997) procedure before and after a negative mood induction. Participants were presented with four different time frames (next week/T1, next month/T2, next year/T3, and next 5 to 10 years/T4) and asked to think of as many events as possible that they were looking forward to or they would enjoy. Participants were randomly allocated to two time periods before the negative mood induction (e.g., next week, next month) and two time periods after the negative mood induction (e.g., next year, next 5–10 years) such that all four time frames were completed by each participant. The presentation of time periods order was counterbalanced across participants (e.g., T1-T2-T3-T4; T2-T1-T4-T3; T3-T4-T1-T2; T4-T3-T2-T1, T1-T2-T3-T4…, the first two time periods before the negative mood induction and the second two time periods after the negative mood induction). For each time period, participants were allowed 1 min to produce as many positive events as they could think of. The pre- and post-negative mood induction responses were aggregated separately to yield a total pre- and post-negative mood induction positive future thinking score, respectively.

*Negative and Positive Mood Induction*. Moore and Oaksford (2002) procedure for the negative mood induction was used. This is an adaptation of the Velten mood induction procedure (Velten, 1968) which combines music, reading statements, and a specific request to participants to try to change their mood. Negative statements, such as ‘There have been days when I felt confused, and everything went miserably wrong, and I was powerless to stop it’ were accompanied by sad music (i.e., Alexander Nevsky Suite, Prokofiev’s Russia under the Mongolian Yoke) that played at half speed. The negative mood induction procedure took about 10 min to complete. After the completion of the second positive future thinking task (see 3.3 Design, recruitment and procedure), all participants also completed a positive mood induction procedure which comprised of reading a list of positive statements such as ‘I feel that many of my friendships will stick with me in the future’ and listening to happy music (i.e., Mozart) simultaneously. The positive mood induction procedure lasted approximately 10 min.

*Visual Analogue Scale (VAS) Mood Rating*. Participants were asked to rate their mood in terms of sadness on a 100 mm VAS just before the first positive future thinking task and again immediately after the negative mood induction. They were asked to rate as follows: ‘At this moment I feel...’ and sadness was printed above the 100 mm line which was anchored on a scale of ‘not at all’ to ‘extremely’.

### 2.3 Design, recruitment and procedure

This study adopted a quantitative experimental research design. All study participants were informed that participation was voluntary, and they were free to withdraw from the study at any stage. Participants’ eligibility to take part in the study was assessed through screening calls on Zoom. For the experimental component of the study, eligible participants were invited to the Suicidal Behaviour Research Health Lab (SBRL) at the University of Glasgow.

To be eligible, participants had to be 18 years or older and be able to attend an appointment at the SBRL Health Lab. Those without a suicidal history (i.e., suicidal behaviours and/or suicidal thoughts) were eligible for the control group, and those who had a lifetime history of suicidal behaviours and/or suicidal thoughts were eligible for the suicidal history group. The exclusion criteria for both groups were as follows: (i) not fluent in English, (ii) being imminently suicidal at the time of recruitment, (iii) being actively psychotic at the time of recruitment, and (iv) having a learning disability or cognitive impairment.

Participants who met the eligibility criteria after the screening calls were provided with a survey link via email. This survey link included the questionnaires described above which participants completed a few days before the experimental session.

*Lab visit*. The experimental component of the study lasted approximately 1 h and was conducted by the lead researcher (GK) with each participant individually. Participants first completed a safety plan, VAS mood ratings, verbal fluency task, and the PFT task for the first two time periods (e.g., next week and next month). Second, they underwent the negative mood induction procedure and then completed VAS mood ratings again. Third, they completed the PFT task for the remaining two time frames (e.g., next year and next 5–10 years). Finally, they completed a positive mood induction procedure and then were debriefed about the study. All participants received a £30 Amazon Voucher as compensation for their study completion. Additional compensation was offered for participants who resided more than 25 miles away from the SBRL Health lab.

### 2.4 Statistical analysis

Analyses were conducted using IBM SPSS Statistics version 29.0.2.0. A G*Power analysis recommended that a minimum of 40 participants was needed. The proposed sample size has a power of 0.8 to detect an effect size of at least d = 0.4 (with alpha at 0.05). Descriptive statistics and bivariate correlations were computed to examine the normality and interrelatedness of all variables. Mean scores and standard deviations across the two groups were also reported. ANCOVA and binary logistic regression analyses were conducted to test the main hypotheses. Depressive symptoms were added as a covariate in the ANCOVA as the positive future thinking effect is postulated to be independent of depressive symptoms. A t-test to explore whether any group differences exist in terms of verbal fluency and an ANOVA to test the effectiveness of the mood induction were also performed. There were no missing cases for our continuous variables. We failed to reject the null hypothesis for Little’s MCAR test, which means our data are missing completely at random (*p* = 0.104). Outliers were also checked using the Mahalonobis Distance Analysis. Herein, the Mahalanobis Distance Analysis detected no outlier.

## 3 Results

### 3.1 Sample characteristics

Ninety-five individuals completed screening calls with the researcher via Zoom, 34 of them were not eligible and a further eight did not attend the lab sessions to complete the whole study. Most people declined as they were unable to attend a lab visit due to distance or they found the compensation for study contribution/travel costs to be insufficient (*n* = 15). Seven were not sufficiently fluent in English to complete the study. Four were excluded because they had a learning disability or cognitive impairment, such as dyslexia. Two were deemed not suitable as they were under the influence of alcohol, and six were experiencing a psychotic episode. Recruitment took place between the 16th of June 2022 and the 30th of April 2023. Fifty-three participants completed all components of the study, and the mean age (*SD*) was 28.42 years (*SD* = 10.96). Their ages ranged from 18 to 65 years. Thirty-four of these participants were women and 19 were men (*n* = 53). In the suicidal thoughts and/or suicidal behaviours history group, there were 20 female and 10 male (*n* = 30) participants while the control group (i.e., those with no suicidal thoughts and suicidal behaviours history) consisted of 14 female and nine male participants (*n* = 23). The two groups did not differ in terms of gender (20 and 14 females for the suicidal history and non-suicidal history groups, respectively, *Chi* (1) = 0.66, *ns*) and age distributions (*M* = 30.4, *SD* = 13.5 and *M* = 26, *SD* = 5.5 in the suicidal history and non-suicidal history groups, respectively, *t* (51) = 1.47, *ns*). Forty one percent (*n* = 22) of participants were white and 38% (*n* = 20) were Asian or Asian British, 17% (*n* = 9) were from other ethnic groups and 3.7% (*n* = 2) did not report their ethnicities. In summary, the two groups did not differ in terms of ethnicity (Chi (1) = 4.07, *ns*). Most participants were single (*n* = 43) and heterosexual/straight (*n* = 40). There were no group differences regarding sexual orientation (Chi (4) = 5.85, *ns*) and marital status (Chi (4) = 0.2.91, *ns*). The groups also did not differ in employment status (Chi (3) = 6.2, *ns*) or education level (Chi (4) = 5.27, *ns*). The two groups did differ in terms of medication use and history of psychiatric illness. Thirty seven percent (*n* = 11) of participants in the suicidal history group (compared to 0% in the non-suicidal history group) were taking antidepressants or anxiolytics (Chi (1) = 10.6, *p* < 0.001) and 57% (*n* = 17) of participants in the suicidal history group (compared to 0% in the non-suicidal history group) had a lifetime diagnosis of a mental health disorder (Chi (1) = 19.2, *p* < 0.001).

There were significant positive correlations between pre- and post-negative mood induction positive future thinking (*p* < 0.001). There were also significant bivariate positive correlations among all study variables except positive future thinking *(p* < 0.001; see Supplementary Table S1).

*Verbal fluency*. Before investigating hypothesis 1, we explored whether the suicidal versus no suicidal history groups differed on verbal fluency. A t-test confirmed that there were no statistically significant group differences in terms of verbal fluency (*M* = 35.2, *SD* = 14.2 for the suicidal history group and *M* = 36.4, *SD* = 9.4 for the non-suicidal history group), *t* (51) = − 0.34, *ns.*

*Mood check*. Repeated measures ANOVA confirmed that sadness increased significantly from the pre-to post-negative mood induction, [*F* (1, 51) = 33.32, *p* < 0.001, *M* = 28.8, *SD* = 26.9 for the pre-mood induction sadness and *M* = 47.0, *SD* = 26.6 for the post-mood induction sadness in the whole sample]. The main effect of the suicidal history group (i.e., suicidal history versus non-suicidal history) was statistically significant [*F* (1, 51) = 18.49, *p* < 0.001]. Those with a suicidal history reported significantly higher levels of sadness across the study (pre-mood induction sadness *M* = 39.6, *SD* = 28.8 and post-mood induction sadness *M* = 57.8, *SD* = 25.0) compared to those without a suicidal history (pre-mood induction sadness: *M* = 14.6, *SD* = 15.7 and post-mood induction sadness: *M* = 32.8, *SD* = 21.9). The effect of the time x suicidal history interaction was not statistically significant [*F* (1, 51) = 0.000, *p* = 0.987].

**Figure 1:**
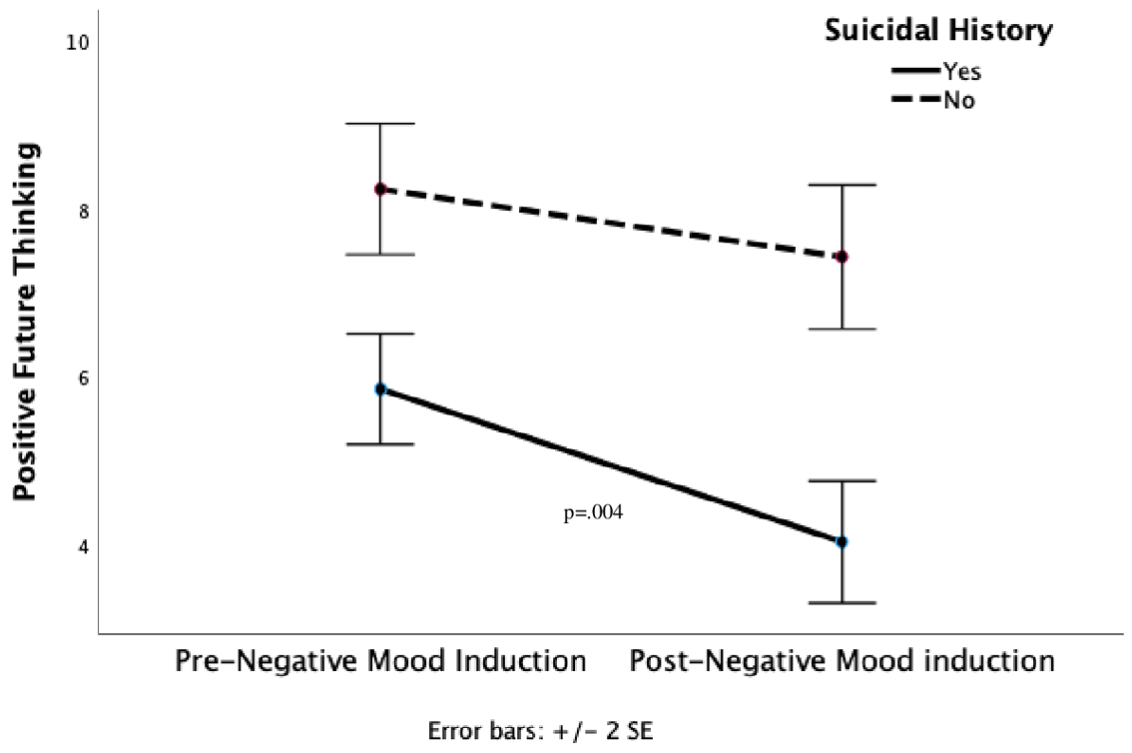
Positive future thinking as a function of suicidal history pre- and post-negative mood induction.

To test hypotheses 1 and 2, a suicidal history group (suicidal versus no suicidal history) x time (pre vs. post) ANCOVA, covarying for current levels of depression, was conducted. It yielded a main effect of suicidal history [*F* (1, 50) = 32.26, *p* < 0.001], with those with a suicidal history reporting significantly fewer positive future thoughts (*M* = 5.0, *SD* = 0.29) compared to those without a suicidal history (*M* = 7.7, *SD* = 0.34). It also demonstrated that mean positive future thinking scores decreased significantly from the pre-to post-negative mood induction [Mean PFT Pre = 6.9, *SD* = 1.95 versus Mean PFT Post = 5.5, *SD* = 2.29; *F* (1, 50) = 13.26, *p* < 0.001]. In addition, there was a significant suicidal history group x time interaction effect [*F* (1, 50) = 4.96, *p* = <0.05]. *Post-hoc* comparisons showed that levels of PFT decreased significantly in those with a suicidal history (pre-mood induction *M* = 5.83, *SD* = 1.53 and post mood-induction *M* = 4.17, *SD* = 1.80; *p* = 0.004), whereas the decrease was not significant among those without a suicidal history (pre-mood induction *M* = 8.26, *SD* = 1.54 and post mood-induction *M* = 7.26, *SD* = 1.57; *p* = 0.078; see Figure 1). Tests of between-participants effects showed that depression [*F* (1,50) = 0.026, *p* = 0.87; as covariate] did not explain a significant amount of variance in PFT measured pre- and post-negative mood induction.

A series of univariate binary logistic regression analyses were conducted to examine hypothesis 3 that individuals with a suicidal history will report higher levels of depression, entrapment, defeat and death-related mental imagery than those without a suicidal history (see Tables 1, 2). As is evident in Table 2, the odds of being in the suicidal history group increased for every unit increase in depression (*Exp(B)* = 0.828, 95% CI [0.734, 0.934], *p* < 0.05), death-related mental imagery (*Exp(B)* = 0.809, 95% CI [0.709, 0.922], *p* < 0.05), total entrapment (*Exp(B)* = 0.731, 95% CI [0.603, 0.887], *p* < 0.05), internal entrapment (*Exp(B)* = 0.606, 95% CI [0.435, 0.845], *p* < 0.05), external entrapment (*Exp(B)* = 0.581, 95% CI [0.415, 0.813], *p* < 0.05), and defeat (*Exp(B)* = 0.739, 95% CI [0.610, 0.896], *p* < 0.05).

**Table 1:** The means and standard deviations of the main psychological factors as a function of suicidal history group.

Variable	With suicidal history M(SD)	Without suicidal history M(SD)	Total M(SD)
PFT 1	5.8 (1.5)	8.3 (1.5)	6.9 (2.0)
PFT 2	4.2 (1.8)	7.3 (1.6)	5.5 (2.3)
Depression	16.1 (6.8)	11.8 (4.1)	15.4 (6.6)
Death-related mental imagery	19.0 (6.8)	12.2 (4.5)	16.1 (6.8)
Entrapment	11.5 (5.2)	6.1 (3.2)	9.2 (5.2)
Internal Entrapment	5.5 (3.0)	3.3 (1.8)	4.8 (2.6)
External Entrapment	6.0 (2.6)	4.2 (1.8)	4.2 (1.8)
Defeat	10.1 (4.9)	5.6 (2.7)	8.1 (4.6)
Suicidal ideation	16.3 (5.7)	9.09 (1.5)	13.2 (5.7)

M, Mean. SD, Standard Deviation. PFT 1, Positive Future Thinking pre-negative mood induction. PFT 2, Positive Future Thinking post-negative mood induction.

**Table 2:** Univariate binary logistic regression analyses for depression, entrapment, defeat and death-related mental imagery as a function of suicidal history (suicidal history vs. no history).

Variable	Beta	SE	95% CI				*p*
			Wald X^2^	LL	UL	OR	
Depression	−0.200	0.064	9.78	0.734	0.934	0.828	0.002
Death-related mental imagery	−0.212	0.067	10.1	0.709	0.922	0.809	0.001
Total Entrapment	−0.313	0.098	10.1	0.603	0.887	0.731	0.001
Internal Entrapment	−0.500	0.169	8.75	0.435	0.845	0.606	0.003
External Entrapment	−0.543	0.171	10.0	0.415	0.813	0.581	0.002
Defeat	−0.302	0.098	9.46	0.610	0.896	0.739	0.002

CI, Confidence Interval; OR, Odds Ratio; SE, Standard Error. Odds Ratios less than 1 indicate increased probability of being in the suicidal history group.

## 4 Discussion

In this study, we experimentally manipulated low mood to investigate its effect on PFT in individuals with and without a lifetime history of suicidal thoughts/behaviours. In addition, we explored the extent to which people with different suicidal histories differed in established suicide risk factors. Overall, our three hypotheses were supported. First, individuals with a history of suicidal thoughts and/or suicidal behaviours generated fewer positive future thoughts (PFT) compared to those without a history of suicidal thoughts or suicidal behaviours (hypothesis 1). In addition, hypothesis 2 was supported. Specifically, we found that after a negative mood induction, the mean PFT scores between the pre- and post-negative mood induction decreased significantly in those participants with a history of suicidal thoughts and/or suicidal behaviours but not in those without a suicidal history. Finally, individuals with a history of suicidal thoughts and/or suicidal behaviours reported higher levels of depression, entrapment, defeat and death-related mental imagery than those without a suicidal history, thereby supporting the third hypothesis.

Our study’s findings are consistent with a classic study by Williams and colleagues; specifically, this earlier study recruited individuals who reported suicidal ideation when depressed in the past (Williams et al., 2008) and used the standard future fluency task and the same negative mood induction procedure as in our study. Their findings were consistent with what we found in our suicidal history group as Williams et al.’ (2008) study showed that participants’ mean scores on positive future thinking decreased after the negative mood induction procedure and hopelessness/suicidality scores were associated with greater declines in positive future fluency from pre to post negative mood induction and lower fluency for positive events following negative mood induction. However, our findings extend theirs as we found that the decrease in positive future thinking is not explained by current levels of depressive symptoms and that the effect is most marked in those with a suicidal history. Crucially, our findings illustrate how even minor fluctuations in negative mood can have a potentially catastrophic effect in those with an existing suicidal history.

The findings related to hypothesis 3 support the key constructs outlined in the IMV model (O'Connor and Kirtley, 2018) and replicate previous research (Souza et al., 2024; O'Connor and Nock, 2014). For example, it is well known that entrapment plays a key role in the pathways that lead to suicidal ideation over time and can even explain the development of suicidal ideation within a few hours (van Ballegooijen et al., 2022). Additionally, as our findings also illustrated, it is well known that significant correlations exist between defeat, entrapment, and suicidal ideation (e.g., Türk et al., 2024). As for death-related mental imagery, individuals with mental images of suicide have more intense suicide ideation in comparison to individuals with no mental images of suicide (Ng et al., 2016) and have a greater preoccupation with mental images of suicide than with suicide-related verbal thoughts (Hales et al., 2011). Finally, the depression finding is consistent with previous research highlighting the role of depressive symptoms in suicide risk (Peterson, 2003; Isacsson, 2000).

While interpreting our findings, a few limitations should be borne in mind. Although the sample size of this study was adequate, it precluded analyses of the contents (e.g., interpersonal) or the different time periods (e.g., next week) associated with positive future thoughts. Given our modest sample size, it was also not possible to compare those who had attempted suicide to those who had only thought about suicide. Indeed, in the context of the ideation-to-action framework (Klonsky et al., 2016), it would be of interest to explore whether the findings reported here would be similar or different if we compared a suicide attempt group to a suicide ideation group. The outcome of such investigation would have important implications for tailoring interventions. Another limitation is the reliance on self-report to ascertain clinical characteristics including suicidal history. Ideally, the findings of this study need to be replicated with different populations, including older people, adolescents, and children. Future research should also include the assessment or consideration of additional factors, such as self-esteem, problem-solving, hopelessness, social support, and coping while investigating the relationship between the risk of suicide and future thinking. It is also worth noting that those in the suicidal history group had significantly lower levels of positive future thinking before the negative mood induction. So, although the two groups were different pre-induction, the fact that the suicidal group’s positive future thoughts decreased even further post-mood induction reinforces the robustness of the finding, as arguably, they were closer to the statistical floor at baseline compared to those without a suicidal history.

The negative mood induction may also have led to an increase in the levels of rumination. Given that previous research has suggested that inducing rumination may increase positive future thinking (Lavender and Watkins, 2004), it would also be helpful to conduct an induction of rumination rather than inducing mood (O’Connor and Williams, 2014) to explore the specificity of the effect. Obviously, the study lacks ecological validity as we conducted the mood induction procedure in the laboratory therefore future studies assessing mood that is induced by real-world events or situations may be insightful.

Despite these limitations, the current study findings point to a number of implications. The findings yield support for the Integrated Motivational-Volitional model of suicidal behaviour (O'Connor and Kirtley, 2018) and the literature investigating the relationship between future thinking and suicide risk experimentally. Building on these findings, in a large observational study, it would be clinically important to test defeat and entrapment as moderators of the positive future thinking–suicidal ideation relationship. It would also be useful to explore the extent to which future thinking interacts with other motivational moderators not included in this study such as perceived burdensomeness and thwarted belongingness (Van Orden et al., 2010). It may be that high levels of positive future thinking could mitigate the effects of feeling a burden on others, thereby protecting those who are vulnerable. Additionally, this study supports the role of key psychological variables (e.g., death-related mental imagery, entrapment, defeat, and depression) in the development and course of the suicidal process (Taylor et al., 2011; O'Connor et al., 2013). Indeed, we need concerted efforts at individual, community and societal levels to tackle the drivers of defeat and entrapment. The identification of the individual, social and cultural drivers will help inform the development of interventions with the potential to alleviate their devastating effects (O'Connor et al., 2023). Clinically, it is important to monitor changes in positive future thoughts in people with an established suicidal history. As such changes may lead people to feel that they have fewer reasons for living and a greater sense of entrapment. However, it is noteworthy that positive future thinking was not correlated with other variables including entrapment and suicidal ideation. This may reflect the fact that positive future thinking is hypothesised to have a moderating rather than direct effect on the entrapment to suicidal ideation relationship, with the former being masked in the correlations. Consequently, improving positive future thinking on its own may not change levels of entrapment and suicidal thinking.

In addition, this is the first study to have included a direct comparison of individuals with and without a suicidal history in terms of positive future thinking using an experimental mood induction procedure. Nonetheless, future experimental work is needed to further disentangle the nature of the association between positive future thinking and suicide risk. Finally, it would also be beneficial for future research to assess the effectiveness of an intervention aimed at improving positive future thinking in individuals who are suicidal. This could build upon pioneering work conducted by Walsh (1993), van Beek et al. (2009) and De Jaegere et al. (2023). In the latter study, De Jaegere et al. (2023) tested future-oriented group training for individuals with suicidal thoughts and found that those who received the intervention exhibited increases in future-oriented thinking at follow-up.

To conclude, participants who reported a suicidal history compared to those without such a history exhibited a dearth of positive future thinking. Positive future thinking was affected by minor mood fluctuations, as those with a history of suicidal thoughts and/or suicidal behaviours showed a significantly more pronounced deterioration in positive future fluency following negative mood induction than those without a history of suicidal thoughts or suicidal behaviours. Participants with a history of suicidal thoughts and/or suicidal behaviours also reported higher levels of depressive symptoms, entrapment, defeat, and death-related mental imagery.

## Data Availability

Data will be made available upon reasonable request from the first author. Requests to access the datasets should be directed to 2325158K@student.gla.ac.uk or goncakose@akdeniz.edu.tr.
