# Supplementary material for: The relationship between positive future thinking, entrapment, defeat and death-related mental imagery in individuals with and without a suicidal history: an experimental study
Source: Front Psychol. 2025 Jun 4;16:1574315. doi: 10.3389/fpsyg.2025.1574315 (PMC12174116; doi:10.3389/fpsyg.2025.1574315)
Supplement: Supplementary file 1 [file Table_1.DOCX]

**Supplementary material**

**Table S1.** Correlations for Study Variables

**1 2 3 4 5 6 7 8**

|  |
| --- |

**1** .682** .048 -.064 -.272 -.052 -.187 -.178

**2** .734** .291 .226 .050 .243 -.080 -.062

**3** -.057 .069 .737** .787** .722** .696** .649**

**4** .043 .009 .731** .652** .863** .869** .660**

**5** -.019 .141 .700** .723** .759** .676** .622**

**6** -.095 -.034 .776** .830** .865** .822** .646**

**7** .109 .079 .645** .628** .569** .551** .786**

**8** -.031 .063 .816** .877** .766** .834** .654**



| *Note.* *******p*<.001, **1** Pre-Positive Future Thinking (PFT), **2** Post PFT, **3** Suicide ideation, **4** Internal entrapment, **5** External entrapment, **6** Defeat, **7** Death-related mental imagery, **8** Depression. Values ​​below the diagonal for participants with a history of suicidal thoughts/behaviours and values ​​above the diagonal for participants without a history of suicidal thoughts/behaviours |
| --- |
